# The relationship between university students’ future work self and career decision-making difficulties: the mediating role of learned helplessness and the moderating role of social loafing

**DOI:** 10.3389/fpsyg.2026.1918115

**Published:** 2026-09-29

**Authors:** Haiming Li, Xingwei Wang

**Affiliations:** 1 College of Telecommunication and Electronic Engineering, Qiqihar University, Qiqihar, Heilongjiang, China; 2 Student Affairs (Student Affairs Office, Armed Forces Department), Qiqihar University, Qiqihar, Heilongjiang, China

**Keywords:** career decision-making difficulties, future work self, learned helplessness, mediation, social loafing

## Abstract

**Background:**

University students often report notable career decision-making difficulties in competitive employment environments. Research has established a positive link between an individual’s future work self and their career development, while learned helplessness and social loafing relate to greater career decision-making difficulties. This study examines the associations among future work self, learned helplessness, social loafing, and career decision-making difficulties, focusing on the mediating role of learned helplessness and the moderating role of social loafing.

**Methods:**

611 university students responded to the Future Work Self Scale, Learned Helplessness Scale, Social Loafing Tendency Questionnaire, and Career Decision-Making Difficulties Scale. The analysis used SmartPLS 4.0 with partial least squares structural equation modeling (PLS-SEM).

**Results:**

Future work self was negatively associated with career decision-making difficulties (*β* = −0.571). Learned helplessness partially mediated this relationship (*β* = −0.055), and social loafing significantly moderated the association between future work self and learned helplessness (*β* = 0.290). The model explained 45.3% of the variance in career decision-making difficulties and 48.7% of the variance in learned helplessness.

**Conclusion:**

The findings indicate that future work self relates to lower levels of learned helplessness and career decision-making difficulties, while higher social loafing corresponds to a weaker association between them.

## 1 Introduction

Research has shown that the economic crisis triggered by the COVID-19 pandemic continues to have lasting effects (Chen and Yeh, 2021). Data released by the International Labour Organization (2025) reveal that the overall unemployment level across the globe held steady at about 5% in 2024, the youth unemployment rate (12.6%) was 2.5 times higher, highlighting the vulnerability of young people during economic recovery. The employment situation for Chinese university students is similarly challenging. With the large number of graduates each year, university students commonly face employment difficulties (Zhou et al., 2024; Xu et al., 2022).

Beyond external factors such as imbalances between job supply and demand, career decision-making difficulties (CDMD) among university students represent an important internal factor associated with their employment challenges. Emerging adulthood (18–25 years old) is a key stage in which individuals explore and establish their career paths, and during this period they often need to cope with various career-related challenges and difficulties (Uzun and Tortumlu, 2025). Career decision-making represents a complex process involving diverse factors and challenges, especially among university students navigating the transition to early adulthood. Previous empirical evidence indicates that difficulties in career decision-making are commonly experienced within this population (Uzun and Tortumlu, 2025; Zhou et al., 2024; Bi et al., 2023).

Parsons (1908) proposed the person–job fit theory (also known as trait–factor theory), which established one of the earliest models of rational career decision-making. This theory suggests that career decision-making involves three key components: self-knowledge, occupational analysis, and the fit between person and job (Parsons, 1908). Building on this perspective, Gati et al. (1996) organized and described the types of obstacles that individuals may encounter when making decisions. Their work is often regarded as one of the early theoretical foundations for studies on CDMD.

CDMD generally refer to a range of challenging problems that individuals may experience during the process of making career-related decisions, which can be understood as a multidimensional and relatively complex psychological state. The construct describes a state in which, due to factors such as insufficient information, internal conflicts, emotional distress, and behavioral rigidity, individuals experience delays and distress in the decision-making process or become dissatisfied with the decision outcomes (Xu and Bhang, 2019; Gati et al., 1996).

Earlier studies have tended to divide the factors influencing CDMD into external and internal dimensions. External support systems include family, society, and environmental resources. For example, mentoring relationships may encourage students to engage more actively in job-seeking behaviors (Kao et al., 2020). In addition, social support, by strengthening psychological capital, may help foster career decision-making self-efficacy, ease uncertainty in career choices, and, in turn, relate to a lower degree of CDMD (Zhou et al., 2024).

Internal psychological mechanisms, such as the future work self (FWS) (Ma et al., 2024; Wang et al., 2024; Lu and Jia, 2022) and proactive personality (Ling et al., 2022; Chan and Chan, 2021), are related to individuals’ decision-making behavior through goal orientation and motivational activation. Aydemir Dev and Bayram Arlı (2025) found that CDMD arise from the interaction between cognitive ambiguity and emotional powerlessness, underscoring the importance of future orientation and control beliefs for decision-making motivation. Their findings suggest that individuals who lack goal clarity are more likely to experience helplessness and decision procrastination, and that ambiguity in career cognition may foster feelings of helplessness, which in turn are associated with employment decision-making difficulties. Therefore, this study explores whether and how the FWS relates to CDMD among university students, using learned helplessness (LH) and social loafing (SL) as guiding perspectives.

### 1.1 Future work self and career decision-making difficulties

From a career decision-making perspective, a deeper issue underlying CDMD concerns the clarity of career identity. University students are in a period of emerging adulthood, during which a central developmental task is to achieve self-identity and a coherent sense of self (Arnett, 2000; Erikson, 1968). Exploring possible career paths and shaping a FWS can be viewed as part of this identity work. The FWS is usually defined as an individual’s imagined ideal work-related identity and reflects how career goals are integrated with self-identity (Strauss et al., 2012).

Prior work suggests that a general future orientation relates to career decision-making outcomes (Diemer and Blustein, 2007; Mulvey and Klein, 1998). Clearer FWS is associated with increased proactive action in career development, higher employability, and stronger career adaptability (Bernabé et al., 2024; Lu and Jia, 2022; Chan and Chan, 2021; Strauss et al., 2012). On this basis, the FWS is frequently discussed as a motivational resource in career decision-making. Building on Strauss et al. (2012), this motivational function is commonly discussed through three mechanisms: Firstly, the function creates a discrepancy between the ideal future self and the current self to energize action. Subsequently, it allows for possibility exploration, facilitating the reconsideration and refinement of one’s self-concept. Additionally, it employs psychological simulation, enabling individuals to mentally project themselves into future scenarios and rehearse potential actions (Zhang et al., 2022; Strauss et al., 2012). Together, these mechanisms have been associated with greater proactivity in career behavior and with lower levels of CDMD.

According to self-regulation theory (Bandura, 1991), the clearer a person’s self-knowledge is, the more easily the self-regulatory system can motivate the individual to invest effort in personal goals and to take corresponding actions. For university students, expectations and aspirations regarding their future work are associated with more active job-seeking activities (Ma et al., 2024; Kao et al., 2020) and greater engagement in career exploration (Lu and Jia, 2022). Lu and Jia (2022) further observed that students with a clearer FWS tend to participate more in exploration activities and report fewer CDMD. Wang et al. (2024) noted that the FWS may function as a motivational resource that promotes individuals’ engagement in proactive career activities, including seeking resources and acquiring new skills. In their study of Chinese nursing interns, this process appeared to facilitate the translation of existing competencies into future-oriented plans and actions. However, the influence of cultural context, such as individualism versus collectivism, may affect how individuals approach career decision-making. For instance, in collectivist cultures like China, career decisions may be shaped more by social roles and group harmony, whereas in individualistic cultures, personal aspirations may play a more dominant role (Pham and Do, 2025). The FWS can transform internal personal resources (e.g., a proactive personality and positive self-evaluations) into the concrete skills, confidence, and initiative needed to cope with the real world of work (Chan and Chan, 2021). Moreover, constructing a clear FWS is a key psychological process through which external support is translated into active job search behavior (Kao et al., 2020).

Recent empirical findings indicate a positive link between the FWS of university students and their employability (Ma et al., 2024). The FWS is also positively associated with university students’ career adaptability, as a clear image of the FWS can stimulate career exploration and encourage students to take on career-related tasks and challenges (Lu and Jia, 2022). Moreover, among Chinese university students, a clearer FWS is linked to better career decision-making, clearer career planning, and fewer career decision-making difficulties (Zhang et al., 2022).

Therefore, university students with a clear FWS are more likely to engage in career exploration and to actively face career tasks and challenges, and they tend to experience fewer difficulties in making career decisions. Accordingly, we propose the following hypothesis: among university students, a clearer FWS is associated with fewer CDMD.

### 1.2 Mediating role of learned helplessness

LH refers to a pattern in which individuals, after repeatedly experiencing situations in which they feel powerless or encounter repeated failure, respond to external stimuli in a passive or indifferent manner (Seligman, 1991). With the expansion of higher education and changes in labor market structures, many university students have begun to experience growing frustration in the job search process (Zhou et al., 2024). In career development contexts, when university students repeatedly face setbacks in job hunting and perceive their efforts as ineffective, this sense of helplessness may gradually become stronger and more firmly established over time (Yeler et al., 2021).

Research suggests that LH may be associated with individuals’ career development through multiple psychological and behavioral mechanisms. For example, those exhibiting elevated LH often interpret unsuccessful outcomes as arising from limited personal competence (Elliott and Dweck, 1988), which can undermine their motivation and willingness to invest effort in career-related tasks (Wigfield, 2024). Empirical studies also indicate that LH substantially constrains university students’ engagement in proactive career behaviors (Uzun and Tortumlu, 2025; Shen et al., 2021). These cognitive and behavioral tendencies together form an important psychological basis for CDMD. Individuals who believe that they lack sufficient ability may avoid employment-related situations and withdraw from career exploration and planning, which in turn is associated with greater difficulty when they need to make career choices. On the basis of this theoretical and empirical evidence, the present study proposes the following hypothesis: among university students, higher levels of LH are associated with more CDMD.

Existing research suggests that a clear career-related self-concept can foster individuals’ proactive career behaviors. Zhao et al. (2025) found that clarity of career self-concept was positively associated with proactive career behavior among Chinese vocational university students. However, the clarity of self-concept is closely linked to individuals’ core self-evaluations. Recent studies have further examined the associations between low core self-evaluations, LH, and job search behavior. In their meta-analysis, Udayar et al. (2020) showed that higher core self-evaluations were linked to lower levels of CDMD. They also reported that individuals with lower core self-evaluations tended to experience less perceived control and greater decision-making procrastination. This pattern may be further shaped by cultural values and self-regulatory factors, such as sleep quality and attentional resources, which are known to influence emotional functioning and decision-making processes in university students (Li et al., 2023). Chen et al. (2023) reported that core self-evaluations were positively associated with job search outcomes through enhanced career exploration and career adaptability. Du et al. (2022) demonstrated that core self-evaluations were associated with career behavior via autonomous life perspectives and career adaptability, highlighting a protective function against helplessness tendencies. Biramo et al. (2025) also observed that job search self-efficacy was positively associated with job search engagement.

Taken together, these empirical findings suggest that individuals with low core self-evaluations are more likely to fall into a LH cognitive pattern. They tend to attribute unemployment and other career-related difficulties to their own insufficient and hard-to-change abilities, which may weaken their perceived employability and, in turn, relate to reduced job search behavior. Based on this body of research, we can construct the following theoretical logic: on the one hand, a clear career self-concept helps to stimulate proactive career behavior; on the other hand, LH arising from low self-evaluations may inhibit individuals’ career exploration and career-related decision-making.

As a future-oriented, career-related self-concept representation, the FWS (Strauss et al., 2012) may be involved in the career decision-making process by shaping beliefs about helplessness. Specifically, when university students lack a well-defined FWS, they may be more prone to LH when encountering career challenges and may doubt their capacity to handle decision-making tasks. This sense of helplessness may, in turn, constrain effective career exploration and planning and be reflected in higher levels of CDMD.

Therefore, we suggest that LH may mediate the association between the FWS and CDMD. More specifically, a more salient FWS is anticipated to correspond with reduced LH and, consequently, fewer CDMD among university students.

### 1.3 Moderating role of social loafing

SL has been characterized as a pattern in which individuals display lower work engagement and invest less effort in group settings than when working alone (Rajaguru et al., 2020; Harkins and Petty, 1982; Latané et al., 1979). Evidence suggests that this pattern is influenced by situational conditions and also reflects a relatively stable propensity—often termed SL tendency—whereby those higher on this propensity typically contribute less effort and show reduced persistence during collaborative tasks (Oneill et al., 2020; Karau and Williams, 1993). This distinction is important for the present study. Although SL is expressed in group contexts, SL tendency reflects an individual difference in how people regulate effort and persistence when personal responsibility is shared or when the contribution of their own effort becomes less salient. Thus, the construct examined here is not a group-level property itself, but an individual tendency toward reduced effort investment under collective conditions. At the university level, cooperative learning is an important instructional format, and the competencies students develop through group interaction are associated with positive outcomes for future career adaptability (Yang et al., 2024). SL not only directly undermines individual effort and motivation, but also disrupts group interaction in learning settings, which can have a substantial negative association with the effectiveness of cooperative learning and with group members’ academic performance (Oneill et al., 2020). In this study, we also treat SL as a behavioral tendency and examine its association with CDMD.

As a future-oriented self-representation, the FWS can, by enhancing goal clarity and career agency, promote career planning and various proactive behaviors; this association has received repeated support across multiple studies and recent reviews (Talluri et al., 2025; Bernabé et al., 2024). From a self-regulation perspective, however, these motivational benefits depend on sustained goal-directed effort. Because SL tendency reflects reduced effort when responsibility is shared or individual contributions are less visible, students higher in SL tendency may be less likely to translate a clear FWS into persistent action. LH functions mainly through impaired self-efficacy arising from perceptions of uncontrollable outcomes. When people expect that their effort is no longer linked to outcomes, they more easily display helpless cognitive and emotional reactions (Wang et al., 2021; Abramson et al., 1978). Thus, reduced effort and fewer mastery experiences or task feedback may weaken the negative association between FWS and LH among students higher in SL tendency.

In team or group tasks, SL can be understood in light of the collective effort model, which proposes that individuals’ level of effort in a group depends on their perceived task value and sense of personal responsibility. When accountability is low, or when individuals believe that their personal contribution is negligible, motivation tends to decline and task avoidance becomes more likely (Kim et al., 2024; Yildiz et al., 2023; George, 1992). Researchers have found that individuals with a high tendency toward SL often show low self-efficacy, low achievement motivation, and a weak sense of responsibility (Mulvey and Klein, 1998), characteristics that are highly consistent with the psychological mechanisms of LH. Accordingly, although career self-regulation is primarily an individual process, the relatively stable effort-regulation tendency reflected in SL may influence how effectively future-oriented career goals are accompanied by persistence and perceived control.

Based on this reasoning, we can infer that even when individuals hold a clear FWS, a high tendency toward SL or exposure to loafing-inducing situations may make it difficult to activate the internal transformation mechanisms through which the FWS is translated into actual effort and a renewed sense of control. Consequently, the capacity of the FWS to relieve LH may be reduced. By contrast, when SL is low—or when evaluability and task significance are high—the FWS is more likely to be translated into effort and perceived control, which may more effectively attenuate helpless tendencies and, ultimately, be associated with fewer CDMD (Strauss et al., 2012; Karau and Williams, 1993; Abramson et al., 1978).

Accordingly, we propose that SL moderates the negative link between the FWS and LH. In particular, under high SL, the buffering effect of the FWS on LH appears weaker, whereas under low SL, this buffering effect appears stronger.

### 1.4 The present study

In sum, with advances in artificial intelligence and other emerging technologies, careers appear more volatile and career choice has become a dynamic process, which can make well-informed decisions more challenging. Students in emerging adulthood seem particularly prone to experiencing CDMD. Against this backdrop, the present study examines how the FWS relates to CDMD among university students and considers the roles of LH and SL in these associations. Our goal is to clarify the mechanisms through which greater clarity of the FWS may correspond to more informed career decision-making and, ultimately, to better employment outcomes.

Hypotheses (see Figure 1):

**Figure 1:**
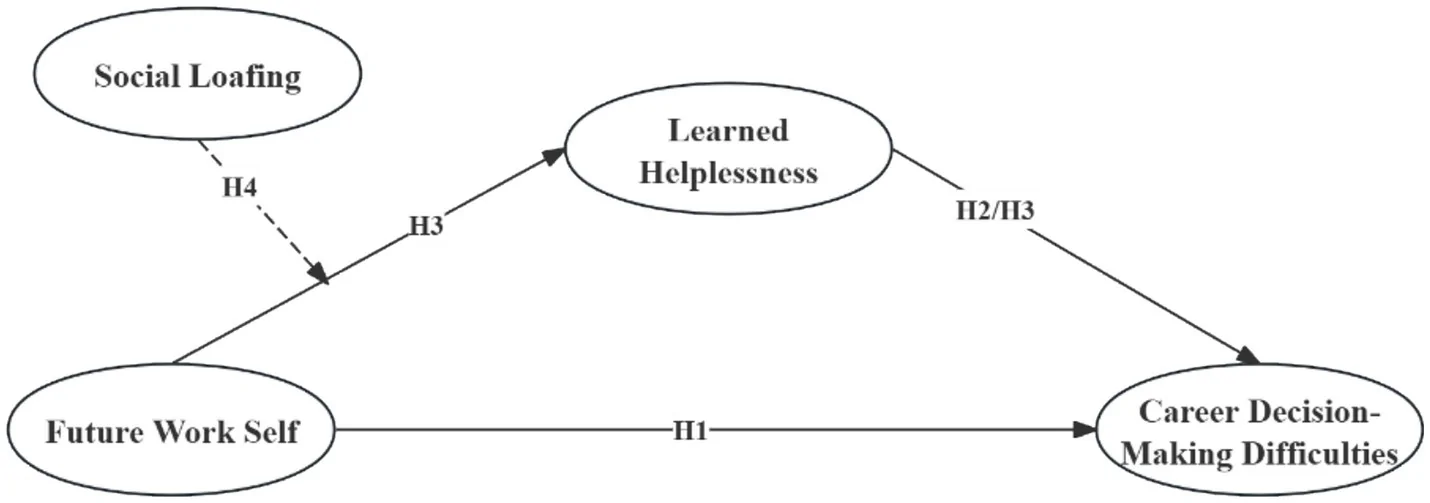
Research model.

*H1*: The FWS is negatively associated with CDMD among university students.

*H2*: LH is positively associated with CDMD among university students.

*H3*: The association between the FWS and CDMD is mediated by LH.

*H4*: SL moderates the negative association between the FWS and LH.

## 2 Methods

### 2.1 Sample and data collection

The investigation took place from May to June 2026 to examine links between the FWS and CDMD among university students. We used a questionnaire survey with simple random sampling within the participating classes. Before the survey was administered, we met with course instructors to describe the study purpose and data collection procedures, and asked them to introduce the background and significance in class to invite participation. A list of eligible students was then obtained from each class, and each student was assigned a numerical identifier. Participants were selected using a lottery or an electronic random-number generator. The questionnaire link was sent individually only to the selected students rather than being posted openly in class group chats. Participation was voluntary and confidential, and students were informed of their right to withdraw at any time. Students provided informed consent before completing the survey.

During data collection, we obtained a total of 643 responses. After excluding invalid questionnaires, such as those completed in less than 1 min (Wang et al., 2024), 611 valid cases remained, yielding an effective rate of 95.02%. The sample included 277 male participants (45.3%) and 334 female participants (54.7%), and most were between 18 and 22 years old (86.91%). Third-year students accounted for 49.4% (302 participants), first-year students for 23.7% (145 participants), second-year students for 13.1% (80 participants), and fourth-year students for 13.7% (84 participants). The majority of participants came from rural areas (75.5%). Table 1 reports descriptive statistics.

**Table 1:** Participants’ demographic characteristics.

Demographic characteristics	Category	Quantity	Proportion
Gender	Male	277	45.3%
	Female	334	54.7%
Age	18–20	216	35.35%
	21–22	315	51.6%
	> 22	80	13.1%
Grade	Freshman	145	23.7%
	Sophomore	80	13.1%
	Junior	302	49.4%
	Senior	84	13.7%
Household registration type	Urban	150	24.5%
	Rural	461	75.5%

### 2.2 Measurement tools

We assessed university students’ CDMD using the Career Decision-Making Difficulties Scale (Du and Long, 2006). The scale comprises 16 items, for example, “I take the initiative to ask others about career information” and “I know what I should do to achieve my career goals.” Previous research has shown that this scale has good reliability and validity (Zhou et al., 2024). The scale comprises four dimensions: career self (5 items), career goal clarity (4 items), career information (3 items), and career planning (4 items). Specifically, low scores on career self-exploration reflect insufficient self-knowledge relevant to career choice; low scores on career information exploration reflect a lack of occupational information; low scores on career goal clarity reflect difficulty forming clear preferences and decision criteria; and low scores on career planning exploration reflect limited preparation for translating preferences into action. These four dimensions thus correspond conceptually to CDMD domains concerning self-information, occupational information, decision-process information, and readiness for decision implementation. This is not intended to reproduce the full Gati taxonomy on a one-to-one basis, but rather to operationalize selected CDMD domains through the absence of career decision-making resources. This operationalization is consistent with the original scale development by Du and Long (2006), who designed the measure within the CDMD framework. Responses are given on a 5-point Likert scale ranging from 1 = “completely inconsistent with me” to 5 = “completely consistent with me.” Although the items are positively worded, lower levels of career self-exploration, goal clarity, career information, and career planning indicate greater difficulties in the career decision-making process. In the original scoring system, higher scores indicate fewer career decision-making difficulties. To align the scoring direction with the theoretical meaning of CDMD in the present study, all 16 items were reverse-scored using the formula 6 − X. Thus, higher recoded scores indicate greater career decision-making difficulties. All subsequent analyses and interpretations were based on the reverse-scored items. The overall reliability of the scale reached a Cronbach’s alpha of 0.946.

We measured the FWS with the five-item instrument developed by Strauss et al. (2012) (e.g., “I am very clear about the kind of person I want to be in my future work”). Prior work with Chinese university samples supports the use of this instrument (Zhang et al., 2022). Responses used a 5-point Likert format (5 = “strongly agree,” 1 = “strongly disagree”); higher scores denote a clearer FWS. Cronbach’s alpha equaled 0.813.

We evaluated LH with the 14-item short form by Lan et al. (2024), derived from Quinless and McDermott Nelson (1988) instrument, which contained 20 items. After validation among Chinese law students, Lan et al. (2024) developed a 14-item version. The version comprises four dimensions—self-doubt (items 1, 9, 11, 12, 13), confidence (3, 4, 14), avoidance (2, 5, 7), and resilience (6, 8, 10). Example items include “When I fail in a task, I tend to attribute my failure to my own dullness” and “I think in most tasks, anyone else could do better than me.” Participants responded to each item on a 5-point Likert scale ranging from 1 = “strongly disagree” to 5 = “strongly agree,” with higher total scores indicating stronger feelings of LH. In the present study, the Cronbach’s alpha for the overall scale was 0.934, and the alphas for the four dimensions were 0.842, 0.747, 0.759, and 0.735, respectively.

We assessed SL using the Social Loafing Tendency Questionnaire adapted by Ying et al. (2014) to measure SL among Chinese university students. This instrument was adapted from the Perceived Social Loafing Questionnaire developed by Høigaard et al. (2010), and its reliability and validity have been confirmed in Chinese samples (Yang et al., 2024). The scale consists of seven items rated on a 5-point Likert scale from 1 (“strongly disagree”) to 5 (“strongly agree”), with higher scores reflecting greater social loafing. Although social loafing is typically manifested in group-performance contexts, the present study does not conceptualize SL as a group-level property or climate. Instead, the questionnaire assesses individual students’ tendency to reduce effort and personal responsibility when working collectively. Research among Chinese university students has also demonstrated meaningful individual differences in social loafing and linked these differences to individual characteristics such as conscientiousness (Yang et al., 2024). Accordingly, SL in the present study refers specifically to an individual-level social loafing tendency expressed in group contexts. In the present study, the scale demonstrated a Cronbach’s alpha of 0.885.

All measurement instruments used in this study were adopted from previously validated Chinese versions. Prior studies have confirmed their cultural adaptability, linguistic equivalence, and psychometric properties in Chinese university student samples. In the present study, reliability and measurement model assessments further supported their suitability for the current participants.

### 2.3 Data analysis

Data analyses were conducted using SPSS 27.0 and SmartPLS 4.0. First, descriptive statistics, reliability analyses, and correlation analyses were performed using SPSS 27.0. Subsequently, Partial Least Squares Structural Equation Modeling (PLS-SEM) was conducted using SmartPLS 4.0 to assess the measurement model and structural model, including the mediation and moderation effects among FWS, LH, SL, and CDMD.

## 3 Results

### 3.1 Descriptive statistics

We conducted descriptive analyses with SPSS using data collected from 611 university students. Table 2 summarizes the descriptive indicators, including means (M), standard deviations (SD), skewness (Sk), kurtosis (Kur), and intercorrelations among the study variables. The absolute skewness values were all below 2 and the absolute kurtosis values were below 7, indicating that the data met the assumption of approximate normality. Correlational analyses further revealed several significant associations among the variables. Specifically, FWS showed a significantly negative correlation with scores on CDMD (*r* = −0.644, *p* < 0.001) and a significant negative correlation with LH (*r* = −0.470, *p* < 0.001), and a significant negative correlation with SL (*r* = −0.394, *p* < 0.001). In contrast, CDMD scores were positively associated with both LH (*r* = 0.433, *p* < 0.001) and SL (*r* = 0.620, *p* < 0.001), suggesting that higher levels of LH and SL were related to greater CDMD. Additionally, LH demonstrated a positive relationship with SL (*r* = 0.537, *p* < 0.001). Overall, these results supported the expected pattern of correlations among the study constructs.

**Table 2:** Descriptive statistics.

Constructs	M ± SD	Sk	Kur	1	2	3	4
FWS	3.023 ± 0.526	0.017	−0.161	1			
CDMD	2.870 ± 0.689	0.244	0.803	−0.644^***^	1		
LH	1.964 ± 0.324	−0.456	−1.043	−0.470^***^	0.433^***^	1	
SL	2.985 ± 0.731	−0.165	−0.485	−0.394^***^	0.620^***^	0.537^***^	1

FWS, Future Work Self; CDMD, Career Decision-Making Difficulties; LH, Learned Helplessness; SL, Social Loafing; ***p < 0.001.

### 3.2 Common method bias

We checked common method bias (CMB) with Harman’s single-factor approach. Results showed that no dominant factor appeared in the analysis (Podsakoff et al., 2003). The first factor explained 38.95% of the variance, remaining below the 50% criterion.

### 3.3 PLS-SEM analysis

Based on the analysis objectives and model settings, PLS-SEM was employed to assess the measurement model and structural model (Sarstedt et al., 2022; Hair et al., 2021). Specifically, this study aimed to examine an integrated model involving indirect associations and a latent interaction effect, while explaining the variance in LH and CDMD. PLS-SEM was retained as the primary estimation approach for two reasons. First, the hypothesized model includes a latent interaction between FWS and SL, and the two-stage procedure provides a direct way to estimate this interaction using latent variable scores (Becker et al., 2018). Second, beyond testing the hypothesized relationships, this study evaluates the explanatory and predictive performance of the model through R^2^ and Q^2^predict, which is consistent with the prediction-oriented capabilities of PLS-SEM (Sarstedt et al., 2022; Hair et al., 2021). Therefore, SmartPLS 4.0 was used to evaluate the measurement model and structural model.

#### 3.3.1 Measurement model

Following Leguina (2015), we evaluated the measurement model in terms of reliability and validity. As shown in Table 3, factor loadings above 0.7 and composite reliability (CR) values exceeding the threshold indicated acceptable reliability. Items with loadings between 0.4 and 0.7 were retained when both CR and average variance extracted (AVE) met the criteria. Convergent validity was confirmed with AVE above 0.5, and discriminant validity was supported by the heterotrait–monotrait ratio (HTMT < 0.85) and the Fornell–Larcker criterion, as all correlations remained lower than AVE roots (see Tables 4, 5).

**Table 3:** Results of the reliability and validity analysis.

Constructs	Items	Outer loadings	Cronbach’s α	CR	AVE
FWS	FWS 1	0.764	0.813	0.870	0.572
	FWS 2	0.716			
	FWS 3	0.776			
	FWS 4	0.759			
	FWS 5	0.764			
CDMD	CDMD 1	0.751	0.946	0.952	0.554
	CDMD 2	0.763			
	CDMD 3	0.753			
	CDMD 4	0.731			
	CDMD 5	0.759			
	CDMD 6	0.760			
	CDMD 7	0.743			
	CDMD 8	0.746			
	CDMD 9	0.727			
	CDMD 10	0.727			
	CDMD 11	0.732			
	CDMD 12	0.762			
	CDMD 13	0.707			
	CDMD 14	0.756			
	CDMD 15	0.712			
	CDMD 16	0.774			
LH	LH 1	0.737	0.934	0.942	0.537
	LH 2	0.721			
	LH 3	0.717			
	LH 4	0.762			
	LH 5	0.764			
	LH 6	0.688			
	LH 7	0.715			
	LH 8	0.705			
	LH 9	0.766			
	LH 10	0.732			
	LH 11	0.703			
	LH 12	0.747			
	LH 13	0.766			
	LH 14	0.730			
SL	SL 1	0.782	0.885	0.910	0.591
	SL 2	0.750			
	SL 3	0.779			
	SL 4	0.750			
	SL 5	0.759			
	SL 6	0.788			
	SL 7	0.773			

FWS, Future Work Self; CDMD, Career Decision-Making Difficulties; LH, Learned Helplessness; SL, Social Loafing.

**Table 4:** Discriminant validity (HTMT criterion).

Constructs	FWS	CDMD	LH	SL
FWS				
CDMD	0.734			
LH	0.540	0.460		
SL	0.460	0.674	0.592	

FWS, Future Work Self; CDMD, Career Decision-Making Difficulties; LH, Learned Helplessness; SL, Social Loafing.

**Table 5:** Discriminant validity (Fornell-Larcker criterion).

Constructs	FWS	CDMD	LH	SL
FWS	***0.756***			
CDMD	−0.655	***0.744***		
LH	−0.471	0.447	***0.733***	
SL	−0.393	0.623	0.543	***0.769***

Bold and italicized diagonal values indicate the square root of the AVE. FWS, Future Work Self; CDMD, Career Decision-Making Difficulties; LH, Learned Helplessness; SL, Social Loafing.

#### 3.3.2 Structural model

We analyzed the structural model through collinearity statistics, path coefficients, and R^2^. Collinearity was tested using the variance inflation factor (VIF), with values below 3.3 considered acceptable (Leguina, 2015). As shown in Table 6, VIF values ranged from 1.183 to 1.286, indicating that multicollinearity was not a concern.

**Table 6:** Collinearity test.

Constructs	FWS	CDMD	LH	SL
FWS		1.286	1.183	
CDMD				
LH		1.286		
SL			1.190	

FWS, Future Work Self; CDMD, Career Decision-Making Difficulties; LH, Learned Helplessness; SL, Social Loafing.

We applied a PLS bootstrapping approach with 5,000 subsamples to estimate path coefficients. Table 7 reports the key findings. The path analysis results showed that FWS was negatively associated with scores on the CDMD scale, indicating that a clearer FWS was associated with fewer career decision-making difficulties (*β* = −0.571, *p* < 0.001), and was significantly and negatively associated with LH (*β* = −0.306, *p* < 0.001). LH was significantly and positively associated with CDMD, indicating that higher LH was associated with greater career decision-making difficulties (*β* = 0.178, *p* < 0.001), while SL was significantly and positively associated with LH (*β* = 0.393, *p* < 0.001).

**Table 7:** Path hypothesis testing.

Hypothesis	Original sample	2.50%	97.50%	T	Results
FWS → CDMD	−0.571	−0.641	−0.499	15.567***	Supported
FWS → LH	−0.306	−0.361	−0.246	10.394***	Supported
LH → CDMD	0.178	0.092	0.263	4.058***	Supported
SL → LH	0.393	0.337	0.452	13.485***	Supported

FWS, Future Work Self; CDMD, Career Decision-Making Difficulties; LH, Learned Helplessness; SL, Social Loafing; ***p < 0.001.

R^2^ and Q^2^ indicate the explanatory and predictive strength of the model (Hair et al., 2021). Table 8 shows R^2^ values reflecting adequate explanation, with predictors covering 45.3% of the variance in CDMD and 48.7% in LH. All Q^2^ statistics were above zero, showing acceptable predictive relevance.

**Table 8:** Explanatory power and predictive relevance (Q^2^predict).

Constructs	R^2^	Q^2^predict	Model fit
CDMD	0.453	0.467	SRMR: 0.048
LH	0.487	0.466	NFI: 0.894

Q^2^ predict represents out-of-sample predictive relevance. “SRMR” denotes the standardized root mean square residual, and “NFI” stands for the normed fit index. FWS, Future Work Self; CDMD, Career Decision-Making Difficulties; LH, Learned Helplessness; SL, Social Loafing.

### 3.4 Mediation analysis

We used the bootstrapping method with 5,000 subsamples to examine the mediating effects among FWS, LH, and CDMD. This procedure let us obtain the indirect effects, the direct effects and their confidence intervals, and we relied on these results to judge the significance and the form of mediation. According to the bootstrapping rule, we judged an indirect effect as significant when its confidence interval did not include zero. The findings showed that LH partially mediated the association between FWS and CDMD. The indirect effect was −0.055 (95% CI [−0.085, −0.027]), while the direct effect was −0.571. Table 9 presents the mediation analysis’s detailed findings.

**Table 9:** Results of the mediation analysis.

Relationship	Indirect effect	2.5%	97.5%	T	Direct effect	T	Type of mediation
FWS → LH → CDMD	−0.055	−0.085	−0.027	3.618***	−0.571	15.567***	CPM

FWS, Future Work Self; CDMD, Career Decision-Making Difficulties; LH, Learned Helplessness; SL, Social Loafing; ***p < 0.001.

### 3.5 Moderating analysis

We tested the moderating role of social loafing on the links among FWS, LH, and CDMD using the two-stage approach in SmartPLS (Becker et al., 2012). As shown in Table 10, SL significantly moderated the relationship between FWS and LH among university students (*β* = 0.290, *p* < 0.001), supporting H4. To further clarify the nature of this moderating effect, we conducted a simple slope analysis. As illustrated in Figure 2, the strength of the relationship between FWS and LH varied across different levels of SL. When SL was low (−1 SD), the FWS was inversely related to LH (the steepest simple slope). This finding indicates that students with low levels of SL and less clearly defined future work selves tend to experience higher levels of LH. When SL was at the mean level, the negative association remained significant but became weaker. When SL was high (+1 SD), the negative association between FWS and LH was significantly attenuated (the flattest simple slope). These results suggest that high levels of SL weaken the protective association between a clear FWS and LH, weakening its ability to protect students from feelings of helplessness. This finding implies that high SL primarily reduces the positive impact of a high FWS on decreasing LH. In other words, when SL is high, even students with a well-defined FWS tend to experience relatively greater LH, indicating that excessive SL can be associated with weaker motivational benefits of a clear FWS.

**Table 10:** Moderating analysis.

Path	*β*	2.5%	97.5%	T	Result
FWS × SL → LH	0.290	0.229	0.346	9.873***	Supported

FWS, Future Work Self; CDMD, Career Decision-Making Difficulties; LH, Learned Helplessness; SL, Social Loafing; ***p < 0.001.

**Figure 2:**
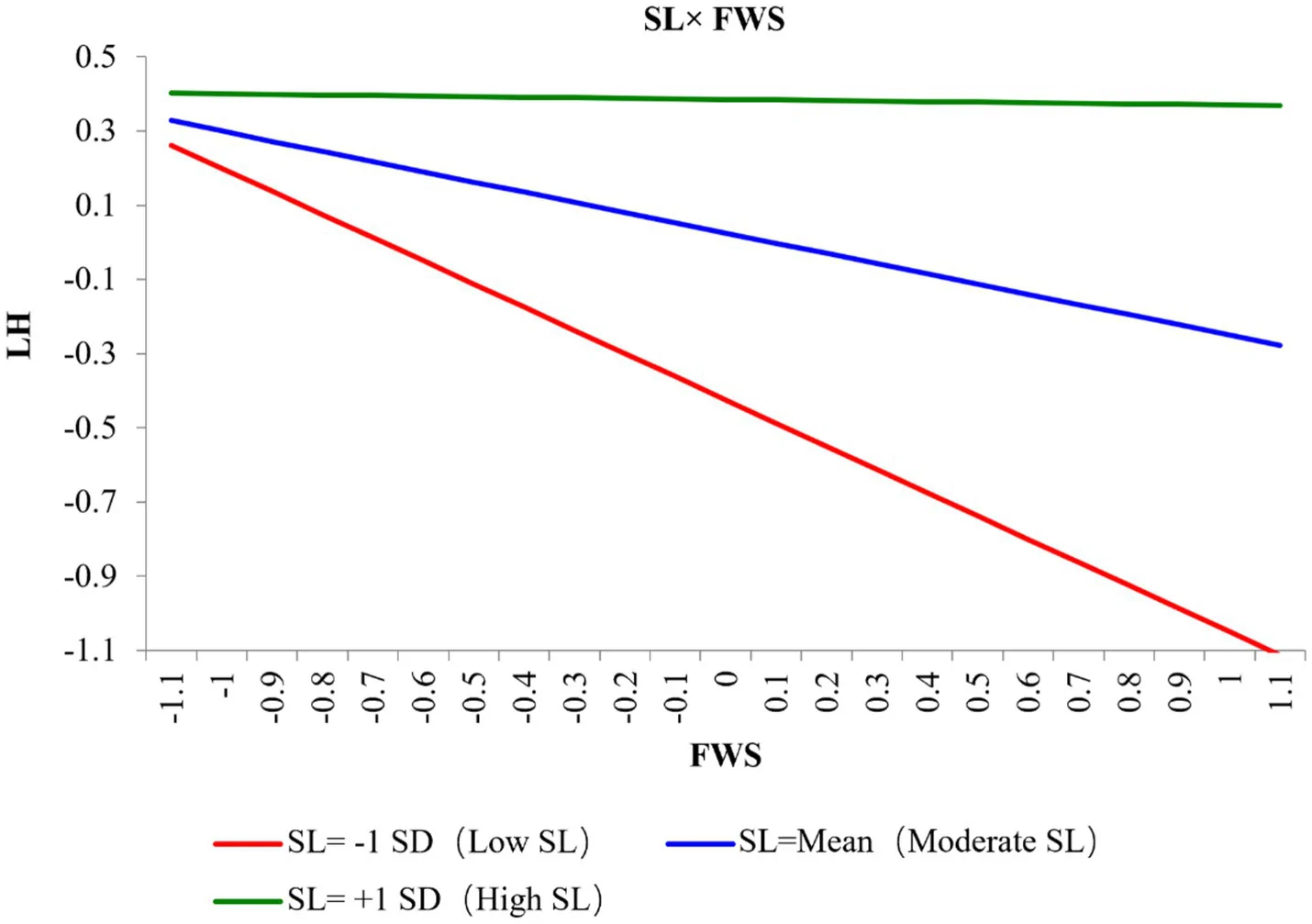
The moderating role of SL in the relationship between FWS and LH.

## 4 Discussion

This study explored the links between FWS and CDMD among university students, with LH as a mediator and SL as a moderator. We found support for all hypotheses: a clearer FWS was associated with fewer CDMD both directly and indirectly, through lower levels of LH. SL significantly moderated the association between FWS and LH. Overall, the model explained 45.3% of the variance in CDMD among university students. These findings not only corroborate the associational role of the FWS but also identify a potential pathway through which these variables are related (via reduced feelings of helplessness) and an important boundary condition (its effect depends on the level of SL). Given the cross-sectional nature of this study, the findings should be interpreted as correlational rather than causal, and no definitive causal conclusions can be drawn. The study enriches the theoretical understanding of the FWS, offers a fresh perspective on the psychological roots of employment-related difficulties among university students, and provides a more fine-grained theoretical basis for targeted career counseling interventions. In the following sections, we discuss these findings in detail in relation to existing literature.

We found a significant negative association between the FWS and CDMD, supporting H1. In this study, higher scores on the CDMD measure indicated greater career decision-making difficulties. When students hold a clearer and more attainable representation of “who they want to become in their future work,” they tend to develop greater goal clarity and career agency. As a result, they tend to show higher levels of proactivity and persistence in key decision-making steps, such as gathering information, comparing alternatives, and executing actions, which is associated with fewer decision-making difficulties and less procrastination (Zhang et al., 2022). This finding is consistent with previous research suggesting that the FWS, as a motivational self-representation, can stimulate proactive career behaviors. By creating a perceived cognitive discrepancy between the current self and the ideal future self, encouraging exploratory behaviors, and promoting psychological simulation, a clearer FWS is associated with more active engagement in career-related tasks and, in turn, fewer CDMD (Lu and Jia, 2022).

From the perspective of self-regulation theory (Carver and Scheier, 1998; Bandura, 1991), the FWS can be regarded as a self-directed system that plays a key role in goal setting and feedback adjustment. When students have a clear vision of the professional they wish to become, they often reflect on their current situation and take steps to move closer to their ideal selves (Lu and Jia, 2022). This process not only strengthens goal alignment but also enhances self-efficacy and a sense of career control (Chan and Chan, 2021). Therefore, the clearer an individual’s FWS, the better they can maintain direction and self-control during complex career decisions. The present study further demonstrates that the FWS serves as a core psychological resource for career development, helping university students transform external employment pressures into intrinsic motivation for action, and being associated with lower levels of CDMD.

This study’s results align with prior research, validating that the FWS acts as a crucial motivational tool that encourages proactive career behaviors and alleviates CDMD. However, this study extends existing research by deepening the understanding of its underlying mechanisms. Unlike prior studies that primarily focused on positive pathways such as career planning (Zhang et al., 2022), career adaptability (Lu and Jia, 2022), and employability (Ma et al., 2024), this study highlights a potential “avoidance” pathway—namely, the FWS may reduce CDMD by alleviating individuals’ LH. This study shows a positive link between LH and CDMD (H2), aligning with learned helplessness theory (Seligman, 1991), which posits that repeated experiences of failure foster a cognitive pattern of perceived helplessness, leading to reduced motivation and behavioral withdrawal. In the career context, university students who experience prolonged job search frustration and lack effective coping strategies tend to develop LH, which discourages them from engaging in career exploration and planning, thereby increasing decision-making difficulties. When students lack a clear FWS, they are more likely to hold maladaptive beliefs that their efforts are ineffective in the face of career pressure and uncertainty (Elliott and Dweck, 1988). Such negative cognitions undermine motivation and promote avoidance behaviors, further exacerbating difficulties in making career decisions (Shen et al., 2021). Uzun and Tortumlu (2025) further emphasized that LH diminishes proactive career behaviors, forming a psychological basis for decision-making difficulties. Therefore, LH can be considered a key correlational variable associated with CDMD.

This study confirmed that LH partially mediates the relationship between FWS and CDMD (H3). This finding not only refines the understanding of the internal mechanism through which the FWS operates but also reveals its psychological protective pathway in the career decision-making process. First, this result deepens our understanding of the motivational function of the FWS. It is not merely a cognitive vision of one’s desired future but a motivational system that regulates individuals’ emotions and attribution patterns when they face career setbacks. In line with previous studies (Biramo et al., 2025; Chen et al., 2023), individuals with a highly salient FWS often possess higher levels of core self-evaluations, leading them to attribute job search failures to temporary or external causes, thereby maintaining positive job-seeking behaviors. In contrast, those lacking a clear FWS tend to have lower self-efficacy and are more likely to generalize or internalize single failures, developing a belief that “no matter how hard I try, I cannot change the outcome”—a hallmark of LH (Du et al., 2022; Udayar et al., 2020). This sense of helplessness undermines individuals’ perceived employability and is associated with avoidance or procrastination behaviors, which manifest as CDMD. Hence, the FWS serves as a facilitative schema, enabling individuals to construct positive meanings from their professional journey. This proactive sense-making mitigates the psychological impact of negative experiences, thereby reducing the formation of helplessness. As a clear and future-oriented career self-concept, the FWS can enhance individuals’ sense of control and self-efficacy, thereby mitigating feelings of helplessness (Strauss et al., 2012). Conversely, when individuals lack a clear sense of their FWS, they are more likely to experience LH when encountering career challenges, which in turn increases difficulties in career decision-making. The FWS is critical in the career decision-making process by alleviating helplessness, as revealed by this mechanism.

At the same time, the findings of this study also support the assumptions of the conservation of resources theory (Hobfoll, 1989). From a resource-based perspective, the FWS can be viewed as a form of psychological resource that helps individuals maintain cognitive stability and goal orientation when facing stress or uncertainty. When this resource is depleted, individuals are more likely to experience LH, thereby triggering a resource loss cycle in their career development. Recent studies have extended this framework to the domain of university students’ career resources. For instance, Antonio and Chiesa (2024) found that courage, as a key psychological resource, encourages students to engage in more proactive career behaviors, thereby effectively reducing their sense of career insecurity. Similarly, Yang et al. (2025) established a model demonstrating that excessive academic pressure depletes psychological resilience, subsequently increasing employment-related anxiety. These findings provide valuable insights for psychological counseling and career education in higher education settings. Strengthening students’ future-oriented awareness and self-efficacy through targeted interventions may effectively prevent the development of LH and support adaptive career decision-making.

In addition, the finding of a partial mediation has important theoretical implications. This indicates that multiple pathways may underlie the association between FWS and CDMD, encompassing both direct and indirect mechanisms. Specifically, the pathway involving LH revealed in this study primarily represents an affective and motivational route. However, the presence of partial mediation strongly suggests that other parallel mechanisms may also contribute to this relationship. For instance, career decision-making self-efficacy, outcome expectations, and perceived employability may serve as additional psychological pathways linking FWS to CDMD (Ma et al., 2024). Individuals with a clear and salient FWS may develop stronger confidence in their career-related abilities, which in turn facilitates more adaptive decision-making behaviors. Future research could further examine whether additional mediating pathways exist between the FWS and CDMD, such as career decision-making self-efficacy, outcome expectations, perceived employability, as well as anxiety, motivation, and volition. For example, Jia et al. (2020) discovered that anxiety acts as a mediator between future time perspective and CDMD. Similarly, the FWS may directly translate into stronger goal commitment and implementation intentions, thereby reducing decision-making difficulties (Strauss et al., 2012).

The results supported the moderating role of SL in the association between FWS and LH (H4). Specifically, the negative association between FWS and LH was stronger among students reporting lower SL and weaker among those reporting higher SL. Although career self-regulation is an individual process, SL reflects how individuals regulate their effort in group contexts and was assessed at the individual level in this study (Yang et al., 2024; Ying et al., 2014). From a self-regulation perspective, a clear FWS may be more strongly associated with lower LH when future-oriented goals are accompanied by sustained effort. One possible interpretation is that, when responsibility is shared or personal contributions are less visible, higher SL may be accompanied by lower effort investment and fewer opportunities for mastery experiences or task feedback. Such conditions could be associated with a weaker perceived connection between personal effort and valued outcomes, which may help explain the weaker association between FWS and LH among students reporting higher SL (Wang et al., 2021). Importantly, mastery experiences, task feedback, effort investment, and perceived control were not directly measured in this study; therefore, these processes should be regarded as theoretical interpretations rather than mechanisms demonstrated by the present data. This interpretation further clarifies why SL is relevant to an individual career context. Although SL is expressed in group activities, it reflects differences in how students maintain effort when responsibility is shared. In collaborative learning or career-preparatory contexts, differences in persistence and perceived control may represent one potential explanation for the observed moderating pattern. Thus, SL can be understood as an individual-level boundary condition rather than a group-level characteristic of career self-regulation. The findings should also be interpreted in light of the demographic composition of the sample. Because 75.5% of the participants had rural household registration, students from rural backgrounds were substantially overrepresented. This imbalance is particularly relevant in the present career-development context because rural and urban Chinese university students may differ in their access to career-related resources. Recent research on Chinese rural college students has identified constraints involving economic resources, educational opportunities, access to high-quality internships, and career-related social networks, together with experiences of employment anxiety and frustration during career development (Wan and Xiong, 2024). Urban–rural differences have also been documented in family cultural capital and access to career-related information and social resources among Chinese university students (Guo et al., 2025; Shen et al., 2021).

These differences provide an important context for interpreting the levels of LH and CDMD observed in the present sample. More restricted access to career information, guidance, and opportunity structures may coincide with greater uncertainty regarding career options and a weaker sense of control over career-related outcomes. Such experiences are conceptually relevant to LH, which is characterized by diminished perceptions of control, and to CDMD, which is closely associated with insufficient information, uncertainty, and reduced confidence in career decision making. Therefore, the observed levels of LH and CDMD should be interpreted in the context of the predominance of rural-background students in the sample. However, the present data do not establish whether rural background is associated with higher levels of LH or CDMD.

### 4.1 Implications

This study integrates FWS, LH, and SL into a holistic model, providing a deeper understanding of the mechanisms behind CDMD. The theoretical contributions of this work are evident in three primary areas. First, it supports the FWS as a significant psychological resource, expanding its theoretical importance in career decision-making (Strauss et al., 2012). Second, it highlights the importance of LH as a mediating variable, echoing the classical discussion of the cognitive mechanisms of helplessness by Abramson et al. (1978). Finally, the introduction of SL as a moderating variable broadens the understanding of how group dynamics shape individual career psychology and behavior, providing a novel viewpoint for future research. It empirically validates the disruptive function of SL within motivational systems, emphasizing the critical role of social and psychological variables in collective learning environments for shaping individual career behaviors. In addition, from a cultural viewpoint, this research presents empirical evidence that sheds light on the unique career behavior patterns of Chinese university students.

Based on the present findings, several practical implications can be derived for university career counselors and educators to reduce LH and facilitate adaptive career decision-making. First, career guidance programs may incorporate FWS–based interventions, such as guided career visualization, structured goal-setting workshops, and narrative career counseling, to help students develop clearer and more attainable career images. For example, counselors and instructors may guide students to reflect on their long-term career aspirations, identify concrete short-term goals, and construct personalized career development plans. These practices may strengthen students’ sense of direction and enhance their intrinsic motivation for career planning, thereby reducing CDMD. Second, psychological counseling services are encouraged to implement targeted interventions aimed at strengthening students’ self-efficacy and attribution patterns. Drawing on cognitive-behavioral approaches, counselors may help students recognize and challenge maladaptive beliefs following academic or job-search setbacks, promote more adaptive attribution styles, and cultivate problem-focused coping strategies. In doing so, such interventions may alleviate LH and foster greater psychological resilience in career-related contexts. Third, given the moderating role of SL, educators designing group-based learning and project tasks should establish clear individual responsibilities, transparent evaluation criteria, and balanced individual–group assessment systems. Providing regular performance feedback and encouraging reflective learning practices may further enhance students’ engagement and persistence. These instructional strategies may help create supportive and accountable learning environments that facilitate the effective translation of FWS into sustained career-related effort. Taken together, these targeted strategies highlight the importance of coordinated efforts among career counselors, psychological service providers, and educators in promoting students’ psychological resources and adaptive career decision-making.

### 4.2 Limitations and future directions

The study has several limitations, which provide opportunities for further improvement in future research. One limitation is that the sample was drawn primarily from Chinese universities, which may limit the generalizability of the findings to other cultural contexts. In addition, 75.5% of the participants had rural household registration. Therefore, the findings may primarily reflect the career-development experiences of rural-background students and may not be fully generalizable to urban students. Future studies could look at cross-cultural comparisons to see if the model applies in different cultures and employ stratified sampling to achieve a more balanced rural–urban distribution, particularly by examining whether the mechanisms linking FWS and CDMD vary between collectivist and individualist cultures and between rural and urban students. This could help clarify whether the associations among cultural values, residential backgrounds, and career-related psychological processes vary across different groups.

Second, the cross-sectional design of this study precludes any causal interpretation of the findings. Future studies could adopt longitudinal tracking designs to examine students’ career development trajectories from enrollment to graduation, or implement experimental intervention studies that manipulate the clarity of the FWS to observe its downstream effects. These approaches would provide stronger evidence for the dynamic pathways proposed in this study. Third, although the SL measure assesses an individual-level tendency, this tendency is specifically expressed in group contexts, whereas FWS and LH were assessed as general individual constructs. Moreover, the study did not directly assess collaborative career-preparation behaviors, effort investment, mastery experiences, or task feedback. Future studies could examine this moderating mechanism within specific group-based career tasks or manipulate individual accountability in experimental designs. Fourth, the current model still contains unexplained variance. Incorporating additional theoretically relevant variables—such as contextual resources (e.g., social support systems, organizational climate, and psychological safety) and individual self-regulatory resources (e.g., sleep quality and attentional functioning), and career decision-making self-efficacy and outcome expectations—may help further illuminate the internal mechanisms underlying individual differences in career decision-making difficulties. Finally, given that the data were collected through self-report measures, there might be an issue with common method bias. Although Harman’s single-factor test was conducted, this approach has inherent limitations and may not fully rule out common method variance. The absence of additional procedural or statistical controls was mainly due to practical constraints related to data collection and research design. Future research could combine behavioral experiments, objective job-search indicators (e.g., number of applications submitted, interview success rate), or peer and mentor evaluations to perform multi-source cross-validation, thereby reducing common method variance and enhancing the robustness and validity of the conclusions.

## 5 Conclusion

Grounded in self-regulation theory, this study constructed and validated a relational model linking FWS, LH, SL, and CDMD. The results showed that the FWS was significantly associated with CDMD both directly and indirectly through LH. Moreover, SL moderated the relationship between FWS and LH. These findings provide theoretical support for understanding the psychological mechanisms underlying university students’ CDMD and offer practical guidance for career counseling and psychological interventions in higher education. Specifically, career counselors and educators may help reduce learned helplessness by incorporating future work self–oriented goal-setting activities and providing structured career planning workshops that emphasize problem-solving and adaptive coping skills.

## Data Availability

The raw data supporting the conclusions of this article will be made available by the authors, without undue reservation.
